# Agmatine Prevents Oxidative-nitrative Stress in Blood Leukocytes Under Streptozotocin-induced Diabetes Mellitus

**DOI:** 10.1515/biol-2019-0033

**Published:** 2019-07-23

**Authors:** Ivanna Bila, Olha Dzydzan, Iryna Brodyak, Natalia Sybirna

**Affiliations:** 1 Department of Biochemistry, Faculty of Biology, Ivan Franko National University of Lviv, Ukraine, 4, Hrushevskyi Str, Lviv 79005, Ukraine

**Keywords:** agmatine, NO-synthase, antioxidant enzymes, leukocytes, streptozotocin-induced DM

## Abstract

Changes in cellular metabolism, development of oxidative-nitrative stress and intensification of glycation and lipid peroxidation (LPO), are significant processes that occur during diabetes mellitus (DM)-associated chronic hyperglycemia. These processes contribute to deviations in the structural organization and functional activity of leukocytes. The development of oxidative-nitrative stress in peripheral blood cells during DM can be prevented by agmatine, an endogenous metabolite of L-arginine, which is a nitric oxide synthase (NOS) inhibitor, and possesses hypoglycemic properties. The administration of agmatine to animals with DM lead to the inhibition of both constitutive and inducible NOS in leukocytes, which in turn decreased total nitrite/nitrate (NOx) levels. Additionally, we observed corresponding increases in reduced glutathione content and activity of antioxidant enzymes (SOD, CAT, GPx, GR), along with decreased levels of the thiobarbituric acid reactive substance, advanced oxidation protein products (AOPPs) and advanced glycosylation end-products (AGEs) as compared to the non-treated diabetic group. Our results indicate that treatment of diabetic animals with agmatine restores redox homeostasis and a balances antioxidant defence system enzymes in leukocytes. This corrective effect on the functional capacity of leukocytes is exerted by preventing oxidative-nitrative stress in animals with DM.

## 1 Introduction

Type 1 diabetes mellitus (DM) is characterized by a progressive autoimmune destruction of pancreatic β-cells, leading to insulin deficiency and chronic hyperglycemia [1]. Some pathways of glucose metabolism that are activated under conditions of hyperglycemia include the autooxidation of glucose and its intermediates to methylglyoxal, glyoxal and 3-deoxyglucosone, which cause non-enzymatic glycosylation of long-lived proteins; the hexosamine pathway producing uridine diphosphate-N-acetylglucosamine which contributes to the glycosylation of serine and threonine residues; sorbitol metabolism, which leads to free radical generation and lower reduced glutathione (GSH) activity; and oxidative phosphorylation leading to mitochondria electron transport chain intensification and the generation of superoxide-anion (O2•−) radicals [2, 3, 4, 5, 6]. The inhibition of glycolysis leads to an accumulation of triose phosphates, which metabolize to carbonyl compounds that take part in oxidative modification of proteins, lipids and DNA. Oxidized proteins activate proteolysis which enhance destructive processes, inflammation and DNA damage, decrease activity of transporter proteins, and alter ATP-ase activity leading to disregulation of the electron transport chain cascade. Furthermore, in presence of Fe^3+^ and Cu^2+^, glucose and its metabolites react with hydrogen peroxide (Н_2_О_2_) forming hydroxyl radical (^•^ОН) to increase oxidative destruction of macromolecules through disruption of any С–Н and С–С bonds [7, 8, 9]. Glucose autooxidation and non-enzymatic glycosylation of amino groups of proteins and phospholipids also leads to an accumulation of metabolism end products (advanced oxidation protein products (AOPPs) and advanced glycosylation end-products (AGEs)) which are difficult to eliminate from the blood and remain in circulation [10]. They are also the source of reactive oxygen species (ROS) since they imitate metal containing oxidation systems. Excessive formation of ROS (O2•−, ^•^ОН, Н_2_О_2_) and reactive nitrogen species (RNS – NO, ONOO^–^, nitroxyl donors) also leads to the development of oxidative-nitrative stress [3, 4, 11]. Therefore, DM is typically characterized by an intensive free radical oxidation of biosubstrates [12]. Simultaneously, the depletion of antioxidant defence mechanisms also occurs during diabetes [13]. A decrease of GSH levels and activity of antioxidant protection enzymes: glutathione peroxidase (GPx), superoxide dismutase (SOD), catalase (CAT), nicotinamide adenine dinucleotide phosphatase and others was observed [5, 14, 15, 16]. These changes produce a favorable background for the formation of atherosclerotic, micro- and macrovascular diabetic complications [11, 17, 18].

The alteration in structural and functional activity of leukocytes is an important part of the pathogenesis of diabetic complications. Changes in intracellular metabolism, intensification of glycation processes and the development of oxidative-nitrative stress in blood cells under conditions of prolonged hyperglycemia, are the main factors which induce pathological changes in the structure of their components and affect their functional state [5, 19]. In particular, changes in the morphofunctional state of leukocytes can be explained by defects in the physiological balance between levels of antioxidants and free radical compounds [20]. The maintenance of intracellular ROS levels within normal limits is crucial for some cytoskeleton-dependent processes, for example, leukocyte migration from the blood into inflammation zones, where they exert their main functions as immunocompetent cells [21]. In our previous research we established [22] that in leukocytes from animals with DM, the total content of actin is reduced, whereas actin polymerization is intensified in membrane cytoskeletal filaments. The development of oxidative-nitrative stress is one of the reasons for the alteration of this dynamic process of actin polymerization-depolymerization in leukocytes during DM. Excessive oxidation of proteins in stress conditions leads to the destruction of F-actin of the cytoskeleton, thereby breaking locomotive and protective functions of leukocytes [22, 23, 24]. The overproduction of ROS and RNS increases the probability of oxidation of thiol groups in tubulin monomers and promotes the formation of disulfide bonds between α- and β-tubulin, which decreases the ability of microtubules to polymerize and alters the structure of actin [25]. The targets of oxidation in F-actin molecules are Cys374 and Met44 residues. The oxidation of Cys374 results in glutathionylation of actin filaments, and oxidation of Met44 leads to the severing of actin filaments [26]. The development of oxidative-nitrative stress in peripheral blood cells during DM can be prevented by endogenous antioxidants. Among a variety of antioxidants, attention will be given to agmatine (4-aminobutyl guanidine), a product of L-arginine decarboxylation [27, 28, 29, 30]. Agmatine inhibits mRNA expression of iNOS and prevents development of oxidative stress under sepsis-induced vascular dysfunction [30]. Furthermore, agmatine alleviates oxidative stress and inhibits the production of cytokines and inflammation under conditions of acute kidney injury in rats [31]. The ability to act as an endogenous nitric oxide synthase inhibitor (NOS) is a distinctive feature of agmatine when compared to other prophylactic and hypoglycemic agents [28, 31, 32, 33]. Since agmatine, as L-arginine, contains a guanidine group it serves as a competitive inhibitor of NOS (Ki = 660 μM for nNOS, 220 μM for iNOS and 7.5 mM for eNOS). Possessing the most potent inhibitory action toward iNOS activity, this amine can be an endogenous regulator of nitric oxide production by iNOS in leukocytes during their activation and functioning in normal and disease conditions [30]. Therefore, arginine decarboxylase which catalyzes the conversion of L-arginine to agmatine, and agmatinase that degrades agmatine, are both present in cells that express cNOS and iNOS [34, 35].

The main aim of our work was to investigate the effects of agmatine on NOS activity and the enzymatic antioxidant defence system, and also to determine the content on nitrites and nitrates, AOPPs, AGEs, GSH and thiobarbituric acid reactive substance (TBARS) of lipid peroxidation (LPO) in leukocytes of peripheral blood of rats under DM.

## 2 Materials and methods

### 2.1 Animal experiments

#### 2.1.1 Induction of diabetes in rats

Wistar male rats with a starting weight of 120–130 g were used for all experiments. Animals were given free access to a standard chow and water and housed under a 12 h light/dark cycle. The room temperature and humidity were maintained were maintained automatically at about 22 ± 2°C and 60 ± 5%, respectively. After several days of adaptation, DM was induced by a single intraperitoneal injection of 6 mg freshly prepared streptozotocin (Sigma Aldrich GmbH, Germany) per 100 g of body weight (b.w.), dissolved in 10 mM Na-citrate buffer (pH 5.5). Animals in the control group were injected with buffer alone. The development of diabetes was detected by the blood glucose measurements made 72 h after the administration of streptozotocin. Animals exhibiting a glucose level above 12 mmol/L were considered included in experiments.

**Ethical approval**: The research related to animals use has been complied with all the relevant national regulations and institutional policies for the care and use of animals.

#### 2.1.2 Experimental design

Animals were divided into four experimental groups: (1) control, (2) control + agmatine, (3) diabetes, and (4) diabetes + agmatine. Starting on the third day following the detection of diabetes, animals from the second and fourth groups were intramuscularly (i.m.) administered agmatine (Sigma, United States) at a daily dose of 20 mg/kg b.w. for 14 days, whereas the animals from the first and third groups were administered saline solution. The compounds were administered daily between 10 and 11 a.m. throughout the experimental period.

### 2.2 Blood collection

After 14 days of treatment, the rats were anesthetized using diethyl ether and euthanized by decapitation. Blood was collected into vials with heparin. Heparin was added beforehand to prevent coagulation (the end point heparin: whole blood dilution = 1:100). 2 ml of blood was centrifuged at 3,000 rpm for 15 min to obtain plasma. Plasma was frozen and stored at –20 °C for further measurements.

### 2.3 Isolation of blood leukocytes

Leukocytes were isolated from blood by centrifugation in over a gradient of ficolltriombrast density (r = 1.076–1.078). Afterwards, the cells were washed twice with cold (4 °C) phosphate buffered saline (PBS: (137 mM NaCl, 2.7 mM KCl, 10 mM Na_2_HPO_4_ × 7H_2_O, 1.8 mM KH_2_PO_4_, pH 7.4)) [36]. Cell viability was measured by trypan blue (0.1 % w/v solution) exclusion test.

Cells (1.5 × 106) were lysed in 250 ul of the buffer composed of 0.5 % Triton X-100, 100 mM KCl, 5 mM MgCl_2_, 2 mM EGTA, 25 mM Tris, pH 7.5 and a protease inhibitor cocktail (Sigma, USA). The lysates of leukocytes were centrifuged at 8,000 × g for 30 min at 4 °C to remove cell debris. Supernatants were then collected and used for biochemical assays.

### 2.4 Determination of NO-synthase activity and measurement of total nitrite and nitrate (NOx) in leukocytes

The production of NO in leukocytes was determined by measuring its breakdown products as nitrate and nitrite (NOx) in deproteinized ethyl alcohol aliquots of lysates. Reduction of nitrates into nitrites was carried out with the addition of vanadium chloride (VCl_3_), which dramatically increased the sensitivity of detection (0.5 μM NO3–). Total nitrite was measured spectrophotometrically at 540 nm using the Grice reagent [37]. The activities of Ca^2+^-dependent (cNOS) and Ca^2+^-independent (iNOS) were determined in lysates of leukocytes in the absence or presence of Ca^2+^ chelator EGTA (4 mmol/L) in the incubation medium instead of CaCl_2_ [38]. L-arginine was added in excess, given its possible utilization by arginase. NOS activity was expressed in nmol NO2– / min × mg of protein.

### 2.5 Antioxidant enzymes assays

SOD (EC 1.15.1.1) activity in leukocytes was determined by the method based on the reduction of nitrotetrazolium by a superoxide radical [39]. The amount of enzyme that inhibits the oxidation of NADH by 50% at 37°C corresponds to one unit (1 U) of SOD activity. The results were expressed as U / mg of protein. CAT (EC 1.11.1.6) activity was measured by decreasing color intensity of the complex formed by H_2_O_2_ with molybdenum salts [40]. One unit of CAT activity was calculated as one nmole H_2_O_2_ / min × mg of protein. GPx (EC 1.11.1.9) activity was assessed by the rate of oxidation of GSH before and after incubation with tertiary butyric hydroperoxide through the colorimetric reaction with 5.5′-dithiobis 2-nitrobenzoic acid (DTNB) [41]. GPx activity was expressed in μmole / min × mg of protein. GR (EC 1.6.4.2) activity was measured by decreasing NADPH absorbance at 340 nm [42]. One unit of GR activity was calculated as one μmole of NADPH oxidized / min × mg of protein.

### 2.6 Protein concentration measurement

The protein content was determined according to Lowry’s method using bovine serum albumin as standard [43].

### 2.7 Determination of malondialdehyde (MDA) concentration, reduced glutathione (GSH) content, AOPPs and AGEs levels

Thiobarbituric acid reactive substance (TBARS) was measured as MDA, and the end product of LPO, in leukocytes and expressed as pmol / million cells with a molar absorption coefficient of 152,000 M^−1^ cm^−1^ at 532 nm [44]. To determine GSH, TCA-deproteinized lysates of leukocytes was used to measure non-protein sulfhydryl compounds which is based on the reaction of GSH with Ellman’s reagent to produce a compound that absorbs at 412 nm. A molar extinction coefficient of 13.600 was used for the nitrobenzoate ion. The concentration of GSH is expressed in nmol / mg of protein [45].

The determination of AOPPs (i.e. some oxidation products with characteristic absorbance) was based on spectrophotometric detection according to Witko-Sarsat et al. [46] modified by Kalousova et al. [47]. The absorbance of the reaction mixture was read at 340 nm. The concentration of AOPPs was expressed in chloramine units (μmol / g of protein). The levels of AGEs in plasma or lysates of leukocytes were determined by measuring fluorescence at an excitation wavelength of 370 nm and emission wavelength of 440 nm [48]. The fluorescence intensity of the samples was expressed as AU / mg of protein.

### 2.8 Statistical analysis

Data were analyzed statistically using Microsoft Excel. Values are expressed as a mean ± standard error of the mean (M ± SEM). The significance of differences between groups was calculated using Student’s *t*-test. P < 0.05 was considered to be statistically significant.

## 3 Results

### 3.1 Effects of agmatine on content of advanced oxidation and glycation end products in rat leukocytes and plasma

**Figure 1:**
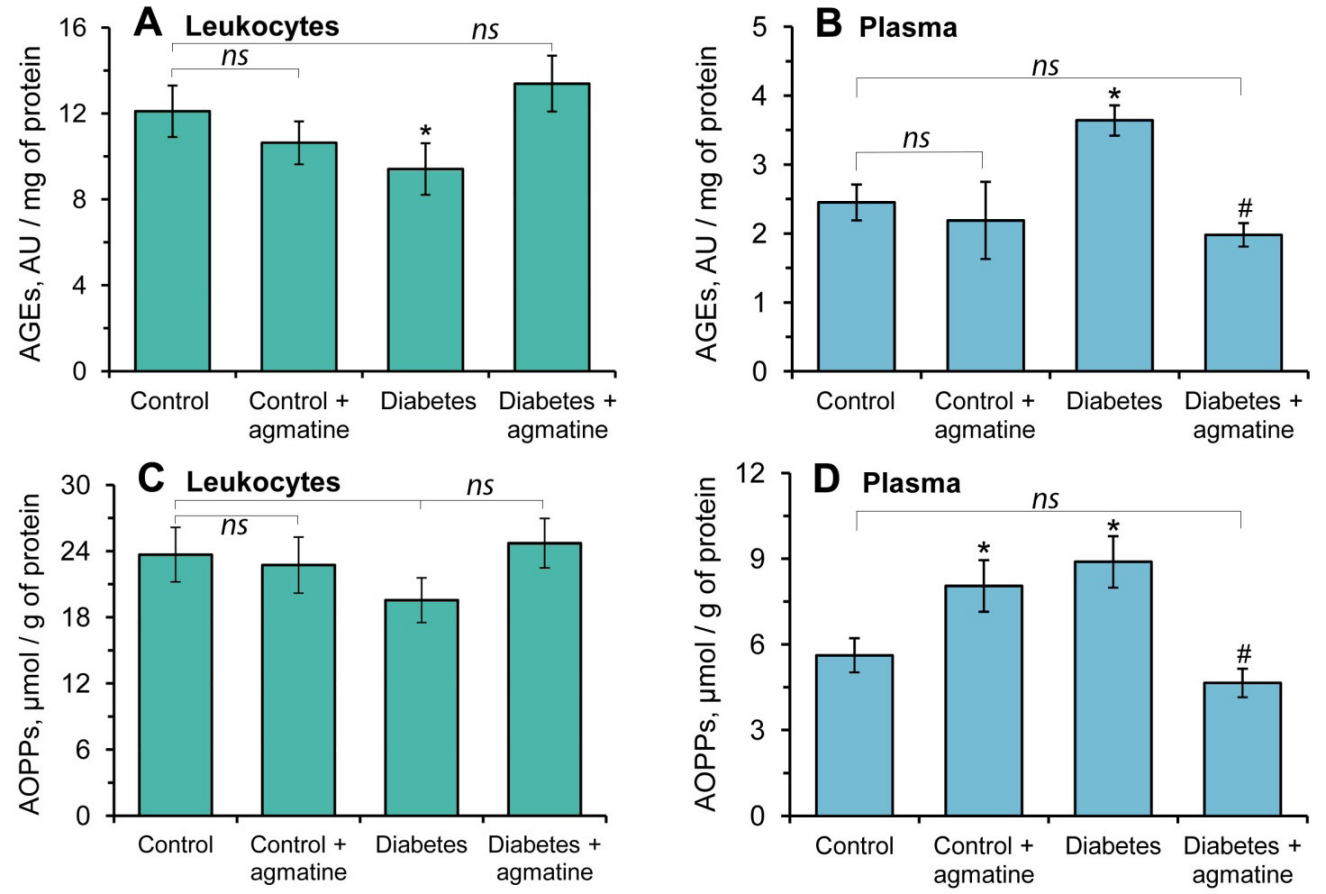
The effect of agmatine (dose of 20 mg/kg b.w.) on the content of advanced glycation end products (AGEs) and advanced oxidation protein products (AOPPs) in blood plasma and leukocytes: (A) AGEs in leukocytes; (B) AGEs in plasma; (C) AOPPs in leukocytes; (D) AOPPs in plasma. The results are shown as the mean ± standard error (n = 8). ns – Not significant compared to the control group (P > 0.05); * – P < 0.05 compared to the control group; ^#^ –P < 0.05 compared to the diabetes group.

We observed a decrease of AGEs in the leukocytes by approximately 22% (Figure 1A) and an increase in plasma AGEs by 1.5-fold during DM when compared to the control group (Figure 1B). There were no significant changes in AGEs levels in the leukocytes and plasma of the control group treated with agmatine (Figure 1A, 1B). In contrast, the administration of agmatine to animals with diabetes caused the decrease of this indicator by 1.8-fold in plasma (Figure 1B), with a coordinate increase in leukocytes when compared to the non-treated diabetic group (Figure 1A).

The plasma AOPPs levels were significantly increased (1.6-fold) in the diabetic group as compared to the control rats (Figure 1D). In addition, there were no significant changes in the AOPPs levels in the leukocytes of diabetes animals when compared to the controls (Figure 1C). After treatment of the control rats with agmatine, the AOPPs levels were significantly increased in plasma as compared to the controls (Figure 1D). The levels of AOPPs did not significant change in the leukocytes of DM rats treated with agmatine when compared to animals with diabetes (Figure 1C). In contrast, the treatment of the diabetes group with agmatine significantly diminished the plasma AOPPs concentration to the control levels (Figure 1D).

### 3.2 Effects of agmatine on NOS activity and total level of nitrite and nitrate in leukocytes

**Figure 2:**
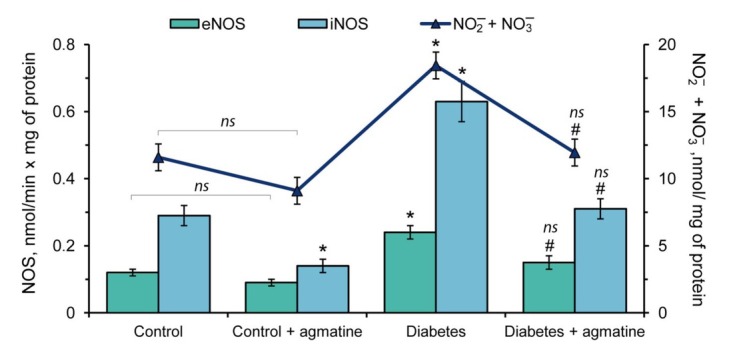
Effect of agmatine (dose of 20 mg/kg b.w.) on activity of different isoforms of NO-synthase (eNOS and iNOS) and total level of nitrite and nitrate in leukocytes of rats. The results are shown as the mean ± standard error (n = 8). *ns* – Not significant compared to the control group (P > 0.05); * –P < 0.05 compared to the control group; ^#^ –P < 0.05 compared to the diabetes group.

The activity of eNOS and iNOS were increased 2.0- and 2.2-fold respectively in leukocytes of rats under DM compared to control. The administration of agmatine to the control animals induced a reduction in iNOS activity (2.1-fold) in leukocytes compared to the non-treated control group (Figure 2). In the agmatine-treated diabetic group the activities of different isoforms of NOS (eNOS and iNOS) were restored to the control levels (Figure 2).

It was found that the total level of nitrite and nitrate were increased 1.6-fold in leukocytes during DM relative to the control group (Figure 2). The administration of agmatine produced a 1.55-fold decrease of these indicators when compared to the diabetes group (Figure 2).

### 3.3 Effects of agmatine on leukocyte lipid peroxidation and antioxidant enzymes

**Figure 3:**
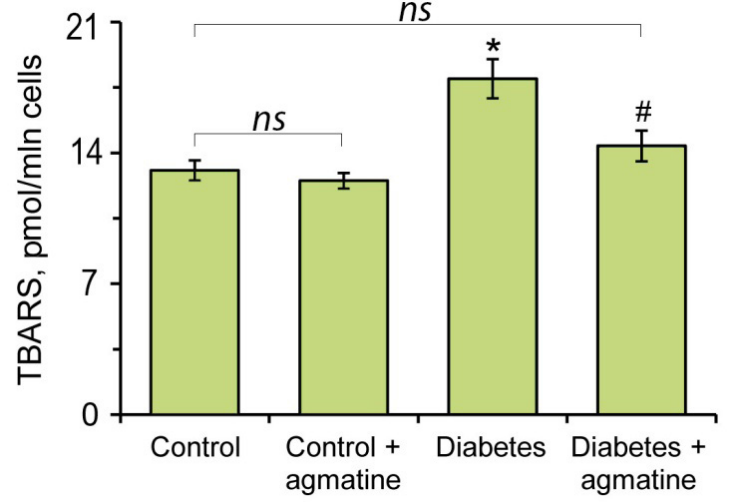
Effects of agmatine (dose of 20 mg/kg b.w.) on the content of TBA-reactive substances (TBARS) of LPO in leukocytes of rats. Results are shown as the mean ± standard error (n = 8). *ns* – Not significant compared to the control group (P > 0.05); * –P < 0.05 compared to the control group; ^#^ –P < 0.05 compared to the diabetes group.

In diabetic animals, the levels of TBA-reactive substances in leukocytes were significantly increased by 1.4-fold as compared to controls (Figure 3). The treatment of diabetes rats with agmatine significantly diminished MDA levels by approximately 20% relative to the diabetic rats, reducing it to control levels (Figure 3).

**Figure 4:**
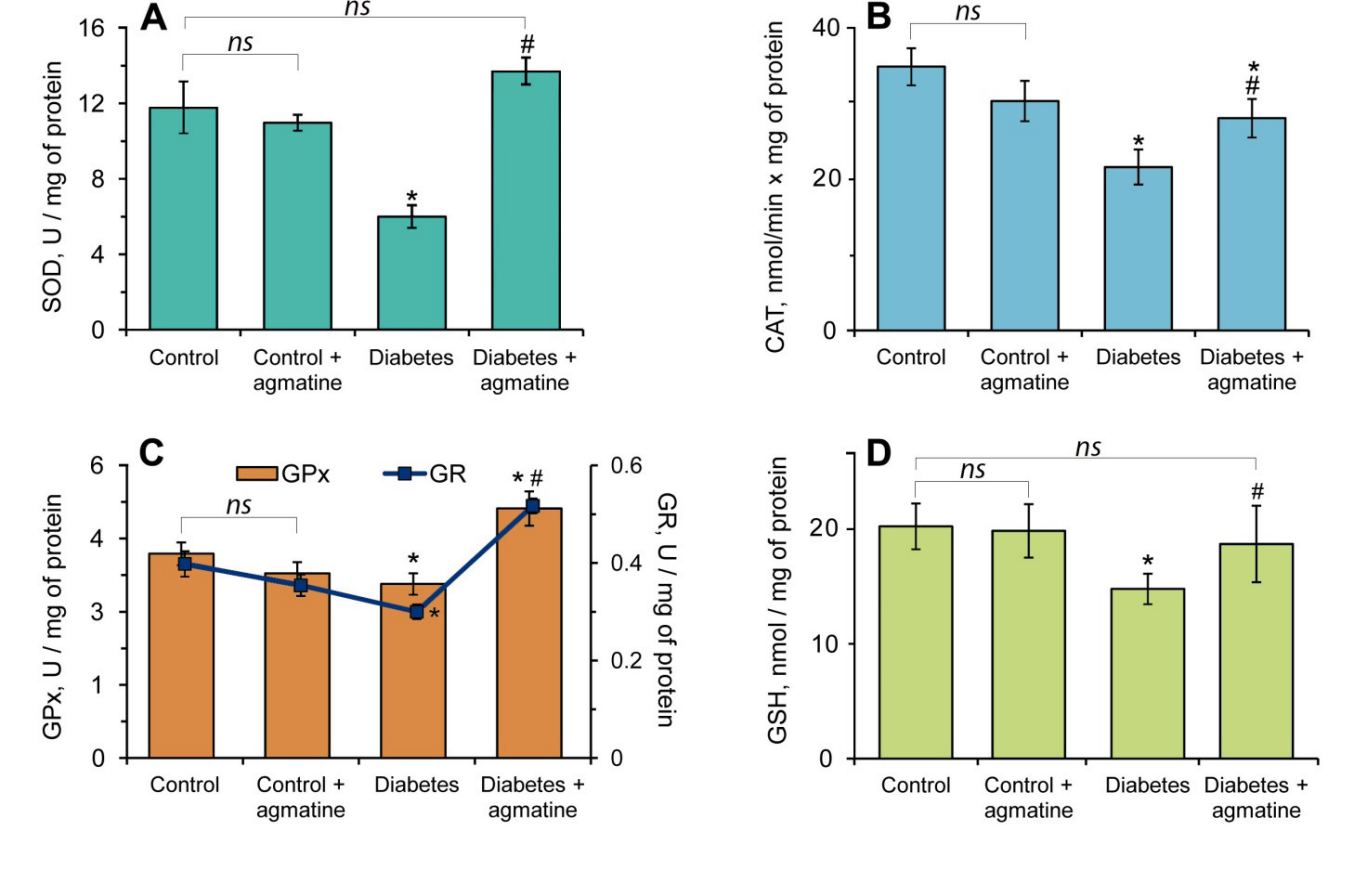
Effects of agmatine (dose of 20 mg/kg b.w.) on activity of SOD (A), CAT (B), GPx (C), GR (C) and level of GSH (D) in leukocytes. Results are shown as the mean ± standard error (n = 8). *ns* – Not significant compared to the control group (P > 0.05); * –P < 0.05 compared to the control group; ^#^ –P < 0.05 compared to the diabetes group.

We observed decreasing SOD activity (1.9-fold) (Figure 4A), CAT activity (1.6-fold) (Figure 4B), GPx and GR activity (1.3-fold) (Figure 4C), respectively, in leukocytes of diabetic animals as compared to the control group. Interestingly, agmatine did not produce significant changes in the activity of antioxidant enzymes in the control group of rats (Figure 4A–C). Administration of agmatine to animals with DM significantly increased SOD (Figure 4A), CAT (Figure 4B), GPx and GR (Figure 4C) activities 2.27-fold, 1.3-fold, 43 % and 72 %, respectively, in comparison to the non-treated diabetes group. In DM, the pool of GSH is exhausted, which is evidenced by a decrease in its content in blood leukocytes by 27 % compared to control (Figure 4D). The administration of agmatine to animals with DM increased this indicator to control levels (Figure 4D).

## 4 Discussion

Leukocytes are a significant source of ROS. Neutrophils and macrophages produce ROS during activation [49]. ROS are involved in leukocyte adhesion enhancement, take part in vasoregulation and fibroblast proliferation. In leukocytes ROS production is also required to maintain physiological functions, including host defense, signal transduction, proliferation, and gene expression of growth differentiation factors and antioxidant enzymes [50, 51, 52, 53]. Numerous studies have confirmed the inducible expression of genes stimulated by excessive ROS production [50, 51, 52, 53]. The source of ROS during DM arises from AGEs, which stimulate the production of excessive proinflammatory cytokines (TNFα, IL-2, IL-6 etc.) [54]. Consequently, in many cell types, and in particular, leukocytes, the expression of iNOS and excessive formation of NO is frequently detected [37, 55, 56]. In this study, we observed increased eNOS and iNOS activity in peripheral blood leukocytes of rats with DM. As a signaling agent, NO is synthesized by eNOS in nanomolar quantities [57]. The approximate 2-fold increase of cNOS activity in leukocytes of diabetic animals leads to excessive formation of NO.

The increase of iNOS activity which is characterized by the production of cytotoxic NO in micromolar concentrations in areas of infection or inflammation provokes additional RNS formation and development of nitrative stress. These changes occur above the background of increasing NO metabolites content (nitrites and nitrates) in leukocytes. The end products of nitric oxide metabolism damage vascular endothelium, disrupt blood circulation and cause the tissue disruption [3, 20]. Under pathological conditions, leukocytes circulate in a subactivated state [58]. The level of ROS production increases by more than 100-fold as a result of NADPH oxidase activity after leukocyte activation. This enzyme catalyzes reduction of molecular oxygen to O2•−. Superoxide-anion reacts with NO at a 3-fold greater rate than with SOD. Therefore, interaction between O2•− and NO occurs with high probability in leukocytes [59, 60]. Disturbance of equilibrium in the direction of O2•− and overproduction of nitric oxide leads to intensive production of cytotoxic ONOO^−^ in leukocytes during DM [20]. Peroxinitrit provokes LPO, enhances sulfhydryl oxidation and causes DNA damage. ONOO^−^ can act on proteins through several mechanisms: binding to metal in active site of enzyme, nitrosylation of protein thiol and amino groups, nitration of tyrosine and tryptophan residues, amino and carboxyl groups of protein and also thiol (cysteine and methionine) and tyrosine oxidation [61]. The 3-nitrotyrosine concentration, which is produced in these reactions, depends on endogenous NO metabolites. Therefore, 3-nitrotyrosine is considered as an indirect marker of peroxynitrite-mediated modifications [62]. The key role in nitration and nitrosylation processes is exerted not NO molecule by itself, but by products of its transformation during the interaction with ROS [63].

We established that agmatine has an inhibitory effect on the NOS activity in leukocytes through decreasing the production of NO. Additionally, we observed the inhibition of cNOS and iNOS in leukocytes of the diabetic animals after the administration of agmatine, which was accompanied by decreased nitrite and nitrate content. Our results also show that agmatine, as competitive inhibitor of NOS, can limit the damaging effects that are mediated by nitric oxide metabolites and diminish the development of nitrative stress during diabetes.

Under diabetic conditions, high levels of glucose in blood and the development of oxidative stress correlates with elevated levels of LPO [64, 65]. Hyperglycemia in conditions of diabetes also increases the rate of plasma AGEs and AOPPs formation. The non-enzymatic modification of plasma proteins may cause deleterious effects, including the generation of ROS and impairment of immune system regulation [54]. ROS affect the lipids of plasma membranes and lead to the formation of lipid radicals (L^•^), which can generate highly toxic lipoperoxid radicals in presence of oxygen (LОО^•^) [66]. The biomarker of LPO intensity is indicated by TBA-positive products. Increased TBA-positive products in blood leukocytes of diabetic animals indicates the activation of lypoperoxidation processes during experimental DM. Disruption of membrane structural-functional integrity, as a consequence of LPO, causes a decreased membrane potential, distortion of transmembrane phospholipid asymmetry and activity of membrane-bound enzymes, receptors and transmembrane proteins and reduced regulatory effect of hormones and mediators [67, 68]. As a result, the functional activity of leukocytes is altered, which becomes a contributor to DM complications [64]. The administration of agmatine to diabetic animals reduced LPO product accumulation in leukocytes. Such an effect is due to its hypoglycemic properties [33], which were confirmed by our previous experimental investigations [28]. The administration of agmatine to diabetic animals caused a reduction of peripheral blood glucose concentration to physiological levels [28], which concomitantly decreased the levels of AOPPs and AGEs in plasma. Under these conditions, overproduction of NADPH-oxidase derived superoxide-anion, xanthine oxidase, glycation and glucose autooxidation products etc. is not detected [69]. Thus, agmatine prevents the development of oxidative stress in blood plasma during DM.

According to our results, the levels of AGEs were significantly decreased in leukocytes of animals with DM. We hypothesize that hypoinsulinemia may reduce translocation of GLUT3 from intracellular vesicles to the plasma membrane in mononuclear leukocytes which has been associated with a decrease in intracellular AGEs formation [70]. On the other hand, the administration of agmatine to animals with DM caused an increase in AGEs levels relative to those of the control group of animals. It is known that activation of I_2_-imidazoline receptors in the adrenal medulla by agmatine enhances the secretion of β-endorphins, leading to activation of μ-opioid receptors on various immune cells (e.g., lymphocytes, neutrophils and monocytes) [33, 71, 72, 73]. Opioid receptors activate mitogen-activated protein kinases and phospholipase C-mediated signalling, thereby leading to the expression or recruitment of the glucose transporters to the membrane [74]. Mechanistically, this could account for GLUT-mediated glucose uptake in leukocytes and the formation of intracellular AGEs [70, 75].

Under diabetic conditions, the intensity of oxidative-nitrative stress and LPO depends on many factors, but in particular, on the orchestrated functioning of antioxidant defence enzymes [3, 20, 76]. The crucial and most potent antioxidant of the first line of defence against ROS, is SOD. Cu-Zn-SOD resides in cytosol, whereas Mn-SOD is primarily found in the mitochondria. These enzymes catalyze the dismutation reaction of two O2•− molecules to form H_2_O_2_ and O_2_ which are less toxic [20]. Hydrogen peroxide is a substrate for a few intracellular enzymes. Catalase, located in peroxisomes, catalyzes the decomposition of H_2_O_2_ into water and molecular oxygen, and glutathione peroxidase reduces it to H_2_O. Reduced glutathione is a donor of hydrogen in this reaction: 2GSH + Н_2_О_2_ → GSSG + 2Н_2_О. Subsequently, oxidized glutathione (GSSG) undergoes reduction by NADPH-dependent GR. Formed NADP^+^ is then restored in the first and third reactions of the pentose phosphate pathway, as well as by the cytochrome chain and a system of natural antioxidants: α-tocopherol, ascorbic acid and flavonoids [67].

From the experimental studies, it was found that the activity of enzymes of the antioxidant defense system is significantly reduced in leukocytes under conditions of DM. We obtained results in antioxidant enzyme activity which are consistent with results of other researchers [39, 77, 78, 79, 80]. The decrease in activity of investigated enzymes can be attributed to a decline in gene expression [81] and oxidation, glycation and nitration processes of protein molecules that exert negative effects on their structure and functional properties. Nitration is accompanied by a change of pKα index of the tyrosine hydroxyl group. This results in negative charging of nitrated tyrosine residues at physiological pH, which alters structural properties of proteins and catalytic activity of enzymes [19]. In our previous studies [29] we showed that content of nitrotyrosine-modified proteins was highly increased under diabetic conditions in leukocytes. In particular, a good example of enzymatic activity loss related to the posttranslational nitration of tyrosine residues *in vivo* is the mitochondrial enzyme Mn-SOD [82]. A decrease of SOD activity produces increased O2•− content in leukocytes that inactivates GR. Thus, during DM, the dysfunction of mutual and cooperative processes, initiated by decreased activity of even one of the antioxidant enzymes due to nitration, glycation and oxidant modification, alters the broader function of the entire antioxidant defence system [20, 83]. A decrease of GSH concentration in leukocytes from rats with DM, is an additional index to confirm the development of oxidative stress and disruption of redox-homeostasis in cellular level [84, 85].

Our experimental data confirm disability of leukocyte antioxidant enzymes to fully implement protective and adaptive mechanisms during conditions of oxidative-nitrative stress, which occurs during the investigated pathology. Administration of agmatine to diabetic animals increased SOD, CAT and GPx activity. Agmatine is known to indirectly cause restoration of flavoprotein oxidases activity (xanthine oxidase, glucose oxidase, monoamine oxidase) to physiological levels, which form hydrogen peroxide in process of biological oxidation [5]. SOD maintains hydrogen peroxide concentrations at a base level for manifestation of antibacterial activity. SOD activity dramatically increases in leukocytes in these conditions. Overproduction of hydrogen peroxide can also damage SOD molecules and disrupt other cell structures [20]. Thus, other enzymes, which decompose hydrogen peroxide, namely, GPx and CAT, are involved in antioxidant protection of leukocytes of diabetic animals. In this regard, SOD and CAT/GPx activities are connected and the level of one enzyme affects the level of other [20].

Administration of agmatine to animals with DM also caused an increase of GSH content. These changes can be accounted for through an increase in GR activity (72% increase relative to DM), which catalyzes the reaction of glutathione reduction. GSH directly inactivates ROS by preventing the formation of highly toxic peroxynitrite [45, 84]. Due to the decrease in the ONOO^−^ concentration, the excessive formation of glutathione radicals (GS^•^) does not occur. This radical does not transform into a prooxidant from antioxidant, so LPO processes return to physiological levels [61]. Thus, in leukocytes from animals with experimental pathology, the administration of agmatine leads to the restored enzymatic balance of antioxidant defense levels.

**Figure 5:**
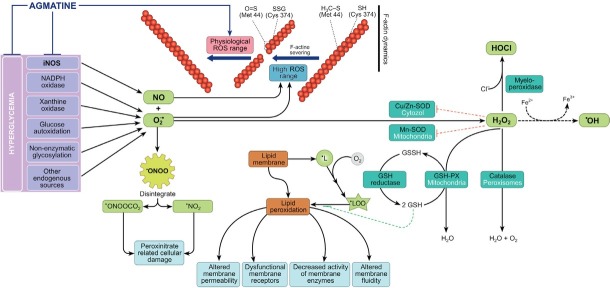
Effect of agmatine on some indices of oxidative-nitrative stress and dynamics of actin polymerization in peripheral blood leukocytes under conditions of diabetes mellitus: іNOS – inducible NO-synthase; NO – nitric oxide; O_2_•− – superoxide-anion radical; ONOO^−^ – peroxynitrite; LPO – lipid peroxidation; GSH – reduced glutathione; GSSG – oxidized glutathione; H_2_O_2_ – hydrogen peroxide; SOD – superoxide dismutase; GPx – glutathione peroxidase; GR – glutathione reductase.

The final analysis of our obtained results is shown on Figure 5. The major mechanism that precedes metabolic changes, enhanced lipid peroxidation, endogenous antioxidant depletion and altered hormonal responses is increased intracellular glucose and downstream metabolic fate [85]. Under hyperglycemic conditions, the excessive formation of O2•− due to glucose autooxidation, nonenzymatic protein glycosylation, intensification of mitochondria electron-transport chain, increase of xanthine oxidase, NADPH-oxidase activity and other metabolic processes and overproduction of NO by iNOS, leads to the development of oxidative-nitrative stress. Superoxide-anion reacts with NO, and cytotoxic ONOO^−^ is formed along with intensification of LPO. The decrease of GSH levels and activity of antioxidant protection enzymes (SOD, CAT, GPx and GR) also occurs [86]. An important role in the downstream biological effects of H_2_O_2_ in leukocytes is the activity of myeloperoxidase. This enzyme catalyzes the reaction of oxygen and halogens to form highly toxic compounds such as hypochlorous acid (Н^+^ + Сl^–^ + Н_2_О_2_ → НОСl + Н_2_О). The resulting hypochlorite causes the chloration of microbial membrane structures that is accompanied by their destruction. These intracellular processes underpin leukocyte antimicrobial activity [87]. The intracellular increase of ROS and RNS concentrations enhance the probability of Cys374 and Met44 residues oxidation in F-actin molecules. This leads to F-actin cytoskeleton destruction, thereby breaking the locomotive and protective function of leukocytes [22, 24, 25, 88]. The production of highly permeable reactive species also causes leakage of these molecules from the phagosomal membrane and deleterious effects on adjacent endothelial cells. In an environment of high oxidative-nitrative stress, these events lead to increased activation of the immune system, damage of endotheliocytes, the development and progression of diabetes complications [89]. Furthermore, the increased levels of cellular reactive species also triggers or enhances programmed cell death [90]. This indicates that an imbalance between continuously produced ROS and RNS and deactivation of these molecules is critical for the maintainence of homeostasis and normal functioning of cell [88, 89, 90].

Agmatine prevents the development of oxidative-nitrative stress in rat leukocytes under conditions of streptozotocin-induced DM (Figure 5). Agmatine also inhibits NO-synthase, and by decreasing NO overproduction in leukocytes, it reduces the intensity of LPO processes, and promotes increased activity of key antioxidant enzymes. Agmatine exerts hypoglycemic and antioxidant effects and maintains ROS and RNS concentrations on physiological levels. This facilitates the recovery of a balance between processes of actin polymerization and depolymerization in leukocytes (Figure 5). A previously described increase in polymerized actin content in the fraction of cytoskeletal filament in leukocytes from animals with DM confirmed this effect [22, 88].

## 5 Conclusions

Agmatine shows an inhibitory effect on NOS activity by decreasing NO overproduction in leukocytes, and also promotes increased activity of key antioxidant enzymes. This antioxidant effect of investigated polyamine is less pronounced in the control group of animals as compared to the DM group. Thus, agmatine, as a compound with hypoglycemic action and NOS inhibitor property, may prevent the development of oxidative-nitrative stress and shows a positive effect on the structural-functional state of blood leukocytes in rats with DM.
